# Strategies to improve fiber utilization in swine

**DOI:** 10.1186/2049-1891-4-11

**Published:** 2013-03-15

**Authors:** Brian J Kerr, Gerald C Shurson

**Affiliations:** 1 USDA-ARS-National Laboratory for Agriculture and the Environment, Ames, IA 50011, USA; 2 University of Minnesota, St. Paul, MN 55108, USA

**Keywords:** Energy, Enzymes, Fiber, Growing-finishing pigs, Nutrient digestibility, Processing

## Abstract

Application of feed processing methods and use of exogenous feed additives in an effort to improve nutrient digestibility of plant-based feed ingredients for swine has been studied for decades. The following review will discuss several of these topics, including: fiber characterization, impact of dietary fiber on gastrointestinal physiology, energy, and nutrient digestibility, mechanical processing of feed on fiber and energy digestibility, and the use of exogenous enzymes in diets fed to growing pigs. Taken together, the diversity and concentration of chemical characteristics that exists among plant-based feed ingredients, as well as interactions among constituents within feed ingredients and diets, suggests that improvements in nutrient digestibility and pig performance from mechanical processing or adding exogenous enzymes to diets fed to swine depends on a better understanding of these characteristics, but also relating enzyme activity to targeted substrates. It may be that an enzyme must not only match a target substrate(s), but there may also need to be a ^′^cocktail^′^ of enzymes to effectively breakdown the complex matrixes of fibrous carbohydrates, such that the negative impact of these compounds on nutrient digestibility or voluntary feed intake are alleviated. With the inverse relationship between fiber content and energy digestibility being well described for several feed ingredients, it is only logical that development of processing techniques or enzymes that degrade fiber, and thereby improve energy digestibility or voluntary feed intake, will be both metabolically and economically beneficial to pork production.

## Introduction

Plant carbohydrates can be classified into three categories: 1) simple sugars and their conjugates (glucose, fructose, etc.); 2) storage reserve compounds (starch); and 3) structural carbohydrates (cellulose, hemicellulose, etc.). Simple sugars and storage compounds are primarily digested in the upper gastrointestinal tract of pigs, although not completely, while structural carbohydrates are only partially degraded by the microflora in the cecum and large intestine [1]. Because most of the starch is removed from corn for ethanol and ^′^sugar^′^ production and from wheat for flour production, resultant co-products (dried distillers grains with solubles-**DDGS**, corn gluten feed, and wheat middlings, respectively) contain concentrated levels of protein, minerals, and fiber [2]. With pigs being able to utilize moderate, but not high levels of fiber in the nursery [3,4] and finisher [5] phases of growth, there is a need to increase the ability of the pig to utilize the energy associated with the structural carbohydrates contained in various ^′^high-fiber^′^ co-products [6]. Due to record high feed prices around the world, it is essential that we find effective ways to minimize the cost associated with meeting the dietary energy and amino acid needs of all livestock and poultry, including swine. In order to accomplish this goal, we need to develop and evaluate technologies that increase digestibility of energy and other nutrients in grain co-products. Use of various processing techniques and exogenous enzymes are two technologies that offer promise for improving the nutritional value of high fiber co-products.

### ″Fiber″ in Swine Nutrition

#### Definition

**Figure 1:**
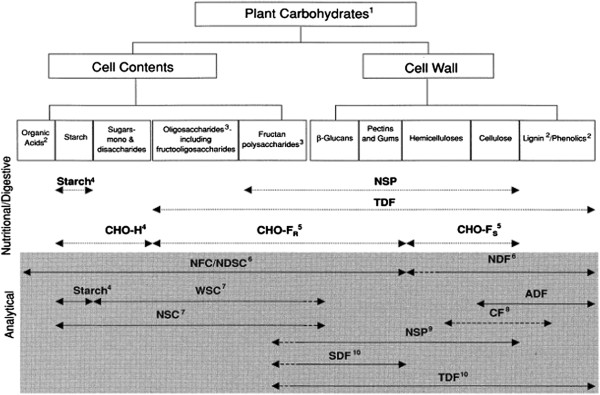
**Nutritional and analytical classifications used to characterize plant carbohydrates [**[7]**].**

Unfortunately, ″fiber″ is perhaps the most poorly understood constituent of swine diets, and is generally described as a complex and highly variable component of plant-based feedstuffs (Figure 1) [7]. It is important to note that the analytical methods used to characterize ″fiber″ often overlap or may exclude fractions of other distinctly different carbohydrate fractions in a feedstuff, and consequently, our ability to adequately relate analytical measures to fiber utilization has been problematic. Some fiber types are more digestible than others, and although they cannot be broken down by mammalian enzymes, they can be fermented by bacteria in the hindgut [8]. These fiber types are often termed ″nonstarch polysaccharides″ (**NSP**), where up to 90% of the cell walls of plants are made up of NSP; of which, cellulose, hemicellulose, and pectins are most abundant [9]. Other less abundant NSP include fructans, glucomannans, galactomannans, mucilages, β-glucans, and gums. Cellulose is found in tightly bound aggregates in plants, while hemicellulose and pectins have sugar side chains that allow them to be more readily broken down. Lignin is not a polysaccharide, but is a high molecular weight polymer, and is not considered a functional dietary constituent because it is indigestible by swine [8]. As shown in Figure 1, common analytical methods used to measure complex carbohydrates in high fiber feed ingredients and feeds include: crude fiber, acid detergent fiber (**ADF**), neutral detergent fiber (**NDF**), soluble and insoluble fractions of total dietary fiber (**TDF**), and NSP. Since each of these fiber methods measures several and sometimes different fractions of complex carbohydrates, they do not adequately relate to the energy value of feeds for swine.

#### Energy value of fiber

The digestibility of ″fiber″ in swine diets can vary dramatically from 0 to 97% depending upon the source of fiber [10], processing method [11], and concentration in the diet [12,13]. However, many NSP are partially fermentable in the hindgut and can be used to produce volatile fatty acids (**VFA**) such as acetate, propionate, and butyrate. These VFA are rapidly absorbed and have been shown to supply between 5 and 28% of the maintenance energy requirement of pigs [14-19]. However, the loss of energy due to methane, hydrogen, and fermentation heat decrease the amount of energy available to the pig from fermentation of fiber in the hindgut [8], thereby decreasing the efficiency of energy utilization [20,21].

#### Fiber alters the gastrointestinal tract

1. Weight

Feeding high fiber diets results in a general increase in the total empty weight of the gastrointestinal tract [12,16,22] and increased gastrointestinal secretions [8]. Jørgensen [23] showed that growing-finishing pigs fed diets containing high dietary fiber (NSP + lignin) (268 g/kg dry matter, **DM**) as compared to pigs fed diets low in dietary fiber [59 g/kg DM), had a significantly heavier stomach, cecum, and colon weights, as well as a longer colon.

2. Enterocyte proliferation

Intestinal epithelial cell proliferation rate is stimulated by feeding high NSP diets [24,25] leading to an increase in cell turnover rate. Growing pigs fed diets containing 10% wheat straw had a 33% increase in the rate of jejunal and colonic cell proliferation, and a 65% increase in cells undergoing cell death [24].

3. Endogenous fluid secretion

The secretion of endogenous fluids is also increased when feeding high fiber diets to pigs [26]. Secretions of saliva, gastric juice, and pancreatic juice were doubled when dietary fiber content was increased from 50 to 180 g/kg in 50 kg pigs [27].

4. Maintenance energy requirement

With the many changes in the characteristics of the gastrointestinal tract due to feeding a high fiber diet, the maintenance energy requirements of pigs may be increased by the extra metabolic demand due the nutrient needs for visceral organ development and maintenance [8,26]. Consequently, methods to improve fiber digestion would reduce these negative effects of fiber on animal metabolism.

5. Gastric emptying and satiety

The rate of gastric emptying may decrease with the addition of certain forms of NSP. Guar gum and pectin increase the viscosity of the digesta [8] and water retention [28]. Growing pigs fed a high energy (starch, casein, soybean oil, and tallow) diet supplemented with 40 to 60 g/kg guar gum had a reduced rate of gastric emptying of 33% to 52% after feeding, and a 27% reduction in DM concentration of the digesta [29,30]. High fiber diets may also contribute to earlier satiety resulting from gastric signals due to the elongation of the stomach wall. Feeding an increased amount of dietary fiber may lead to increased volume of digesta in the stomach, decreased transit time, and increased satiety. This is important in gestating sows because if they are satisfied physically and nutritionally, they appeared to be less stressed and exhibited decreased physical activity [31].

6. Digesta passage rate and nutrient utilization

The passage rate of digesta can also be affected by feeding diets high in fiber. Some studies have shown increasing daily DM flow at the terminal ileum when NDF levels were increased in the diet [32]. Others have also shown up to a 14% and 23% increase in rate of passage when 75 to 300 g of bran or oatmeal co-products, respectively, was added to the diet [33]. These results suggest that the differences in rate of passage through the total digestive tract may be due to differences in the rate of passage through the large intestine, because neither fiber source had a significant effect on gastric emptying or passage through the small intestine [33]. Additionally, particle size of a fiber source may also contribute to the rate of passage. Bardon and Fioramonti [34] showed that a large particle size of wheat bran decreases transit time compared to a smaller particle size.

The amount of time the digestive contents spend in the large intestine can also affect the capacity for fermentation. Fiber fermentation in the cecum and colon results in the production of VFA (mainly acetic, propionic, and butyric acids) which are viable sources of energy. However, the energy density and digestibility of the diet usually decreases with the addition of NSP [8]. In addition, NSP reduces lipid absorption due to a partial inhibition of both lipolysis and intestinal fat absorption [35]. Nonstarch polysaccharides also decrease dietary nitrogen (**N**) retention due to increased secretion of endogenous N, which leads to increased bacterial N excretion [8]. Although minerals do not contribute energy directly to the diet, an impact of NSP on mineral utilization should also be considered (i.e., deficiencies or excesses could lead to physiological conditions that may ultimately affect energy absorption). However, the impact of NSP sources on mineral utilization appears to be minimal [8,36].

### Mechanical Processing Effects on Fiber Utilization

Limited data are available relative to the effect of processing (mechanical or chemical) on changes in fiber utilization in non-ruminants. Teitge et al. [37] reported that pelleting and micronizing, but not steam-flaking, resulted in a greater response to a dietary pentosanase in broilers fed diets containing rye, while Brenes et al. [38] indicated that autoclaving lupins had no impact on chick performance. Autoclaving high-tannin peas, in contrast to low-tannin peas, improved apparent metabolizable energy and apparent protein digestibility in Leghorn chicks [39]. In 80 kg pigs fed barley-based diets, pelleting had no effect on ileal or fecal apparent digestibilities of DM, gross energy (**GE**), crude protein (**CP**), fat, or fiber (NSP + lignin), although it did increase pre-ileal apparent digestibility of starch [40]. In contrast to Teitge et al. [37], Graham et al. [40] reported that pelleting did not improve the digestibility response found when dietary ß-glucanase was added to the diet.

Poel et al. [41] reported that steam processing of faba bean cotyledons did not improve ileal digestibility of CP, either due to the low level of trypsin inhibitor activity present in faba beans, or due to the trypsin inhibitor being sensitive to heat above the 100°C which was used in this study. Likewise, Thacker and Campbell [42] and Nyachoti et al. [43] showed little effect of micronization on nutrient digestibility coefficients. Pelleting of diets containing high levels of corn fiber (corn gluten feed), improved N balance, apparently due to the increased availability of tryptophan [44]. Extrusion is a heat processing method for feed ingredients that is commonly used in the commercial feed industry. However, very little is known about the effects of extruding corn and corn co-products on nutritional value [6]. Additional detailed information regarding the impact of feed processing on energy and nutrient digestibility has been published in reviews by Hancock and Behnke [45] and Stark [46].

### Effects of Exogenous Enzymes on Fiber Utilization

#### Poultry vs. swine diets

The addition of exogenous enzymes to animal feeds in efforts to improve nutrient digestion is not a new concept and responses have been reviewed in detail [47,48]. The majority of commercial enzyme products has been targeted toward poultry [49,50] and are typically added to diets containing barley, oats, peas, rye, or wheat [51-54]. Few studies evaluating enzyme use in corn-soybean meal diets have been published [55].

#### Enzymes in non-corn based swine diets

As with poultry, the majority of research on adding enzymes to swine diets has focused on non-corn-based diets. Adding a multi-enzyme complex to diets containing barley and wheat has been shown to improve soluble NSP digestibility in 10 kg pigs, although growth performance was not affected [56]. Similarly, variation in responses from enzyme addition in pig diets has also been reported by Nonn et al. [57], who found no effect of enzyme supplementation on pig growth performance, even though they observed increased digestibility of crude fiber and cellulose. Likewise, Thacker and Campbell [43] indicated that although enzyme supplementation increased nutrient digestibility coefficients, there was little effect on pig growth performance. In contrast, Omogbenigun et al. [58] supplemented an enzyme cocktail (cellulase, galactanase, mannase, and pectinase) to a wheat-based diet fed in 6 kg pigs and observed an improvement in growth performance (growth rate and feed efficiency) over a 38 d period. Improved nutrient digestibility has also been reported by Yin et al. [59] who added xylanase to diets containing wheat co-products fed to 15 kg pigs and reported improved ileal and total tract apparent digestibility of DM, CP, and energy, especially in diets containing high levels of insoluble NSP. Lastly, adding an enzyme cocktail (fermentation extracts and solubles from *A. niger* and *T. longibranchautum*) to a diet containing 20% soy hulls improved DM and energy digestibility, but not N digestibility, in 33 to 51 kg pigs [60]. With soybean hulls having a large proportion of cellulose relative to other NSP, these data provide some evidence that cellulose digestion can be impacted in addition to hemicellulose and the more soluble forms of fiber.

#### Enzymes in corn-based swine diets

Limited research has been reported on the impact of exogenous enzymes on nutrient digestibility or pig performance when fed corn-based diets. Supplementation of β-glucanase to a corn-soybean meal-based diet had no impact on DM, energy, or CP digestibility in 6 kg pigs [61]. Likewise, supplementation of β-mannanase (β-mannose is a part of hemicellulose) to a corn-soybean meal-based diet failed to show any effect on DM, energy, or N digestibility in 93 kg barrows [62]. However, adding β-mannanase improved feed efficiency in 6 kg pigs (42 d feeding period) and 14 kg pigs (21 d feeding period), and improved gain and feed efficiency, but had no impact on carcass composition, when fed from 23 to 110 kg [62]. Kim et al. [63] utilized a carbohydrase enzyme mixture (α-1,6-galactosidase and β-1,4 mannanase) in corn-soybean meal-based diets fed to nursery pigs and reported an improvement in feed efficiency in two trials (35 d trial, 6.3 to 19.1 kg BW; and a 21 d trial, 8.0 to 15.2 kg BW), as well as an improvement in ileal energy digestibility. Supplementation of the carbohydrase enzyme mixture also decreased the concentration of stachyose in the proximal and distal small intestine, and raffinose concentration in the distal small intestine, suggesting that this carbohydrase mixture improved the digestibility of carbohydrates in soybean meal. In a similar manner, supplementation of several multi-enzyme preparations added to corn and soybean meal-based diets (small amounts of wheat, wheat screenings, barley, mill-run, canola meal, and peas) fed to 7 kg pigs for 28 d, improved growth performance and various nutrient digestibility indices in both the ileum and total tract (Table 1) [58].

**Table 1:** **Effect of enzyme supplementation on growth performance, percent apparent ileal digestibility (AID), and total-tract digestibility (TTD) of nutrients in 7 kg pigs**^**1**^

	**Diet**^**2**^				**Statistics**	
**Performance**	**Control**	**C + Enz A**	**C + Enz B**	**C + Enz C**	**SEM**	**P-value**
ADG, g	224^b^	252^a^	263^a^	249^a^	7.9	0.02
ADFI, g	432	435	456	414	17.8	0.42
G:F	0.52^b^	0.58^a^	0.58^a^	0.61^a^	0.02	0.01
AID,%						
DM	60.1^b^	65.8	66.1^a^	66.7^a^	1.5	0.01
Starch	86.7^b^	92.6^a^	94.6^a^	95.3^a^	1.1	0.02
GE	62.8^b^	70.0^a^	69.7^a^	71.4^a^	0.9	0.01
CP	62.1^b^	71.5^a^	71.4^a^	73.2^a^	1.5	0.01
Phytate	59.2^b^	71.7^a^	69.1^a^	69.7^a^	2.3	0.04
NSP	10.1^b^	14.9^a^	16.4^a^	21.4^a^	1.4	0.01
TTD,%						
DM	75.6^b^	78.1	77.2^a^	80.0^a^	0.5	0.01
Starch	94.4^b^	98.6^a^	97.6^a^	98.6^a^	0.7	0.01
GE	77.8^b^	79.8^a^	79.8^a^	81.1^a^	0.7	0.01
CP	67.1^b^	71.2^a^	71.6^a^	74.2^a^	1.0	0.01
Phytate	69.4^b^	96.8^a^	96.3^a^	96.0^a^	3.2	0.01
NSP	48.9^b^	61.2^a^	59.6^a^	66.8^a^	1.2	0.01

^1^Average initial weight, 7.0 kg, 28 d trial, 6 pigs/trt, ADFI on a DM basis [58].

^2^Enzyme preparations provided 250 units xylanase, 150 units glucanase, 0.001% amalyase, 0.0003% protease, 0.002% invertase, and 400 units phytase per kilogram of diet and differed in the type of plant cell wall degrading activities. Enzyme A contained cellulase, galactanase, and mannanase; Enzyme B contained cellulase and pectinase; and Enzyme C contained cellulase, galactanase, mannanase, and pectinase.

^abc^Means within a row with different superscripts differ at the P-value shown.

Recently, Ji et al. [64] evaluated adding a β-glucanase-protease enzyme blend to a corn-soybean meal diet fed to 38 kg pigs (Table 2). Pigs fed the enzyme blend diet had increased total tract digestibility of DM, energy, CP, TDF, and phosphorus (**P**), but only increased ileal digestibility of NDF, while ileal digestibility of CP appeared to decrease. These authors suggested that the increase in ileal NDF digestibility (and hemicellulose), with no change in fecal digestibility due to enzyme supplementation, may have shifted some of the digestion of these nutrients from the hindgut to the small intestine, which would avoid the fermentative loss of energy and presumably increase the energetic efficiency of fiber digestion.

**Table 2:** **Effect of enzyme supplementation on percent apparent ileal digestibility (AID) and total-tract digestibility (TTD) of nutrients in 38 kg pigs**^**1**^

	**Diet**^**2**^			**Statistics**	
**AID,%**	**Basal**	**B + 0.05%**	**B + 0.10%**	**B vs Enz**	**0.05 vs 0.10**
DM	70.86	69.13	70.50	0.33	0.25
Energy	70.93	69.48	70.71	0.44	0.31
CP	78.29	75.51	76.54	0.04	0.37
Starch	97.95	98.01	98.12	0.51	0.59
NDF	1.21	9.52	10.05	0.02	0.88
ADF	4.33	4.36	5.22	0.91	0.84
TDF	NA	NA	NA	NA	NA
Crude fat	61.40	62.94	62.18	0.49	0.68
P	49.62	49.54	49.00	0.86	0.80
TTD,%					
DM	87.42	88.61	88.50	0.01	0.62
Energy	86.51	87.42	87.26	0.01	0.51
CP	86.47	88.08	87.39	0.01	0.10
Starch	99.24	99.26	99.31	0.53	0.44
NDF	54.62	55.62	56.05	0.36	0.77
ADF	64.84	61.40	65.92	0.40	0.01
TDF	60.61	65.36	65.61	0.01	0.86
Crude fat	80.14	80.51	78.24	0.51	0.09
P	53.80	61.73	57.83	0.01	0.01

^1^Average initial weight, 38.2 kg, 4×4 Latin Square with 14 d periods (4 d adapt, 5 d fecal collection, 3 d transition, 2 d ileal collection) [64].

^2^Enzyme contained 660 β-glucanase units/g and 22 hemoglobin units/g.

#### Enzymes in swine diets containing DDGS

Spencer et al. [65] reported that adding an enzyme preparation to diets containing 30% DDGS increased growth performance in nursery pigs. However, the potential benefits of adding enzymes to diets containing increasing levels of corn co-products on growth performance of finishing pigs remains largely unknown.

Recently, we completed an extensive apparent total tract digestibility and performance trial with enzyme/feed additives commercially available in the United States [66]. In general, the enzymes contained glucanase, hemicellulase, and/or xylanase activities, the probiotics contained either *Pediococcus* or *Bacillus* activities, while the yeast product was derived from *Saccharomyces*. In general, the products were selected based on their potential to affect energy and fiber digestion, or their ability to modulate the bacterial ecology within the gastrointestinal tract. Basal diets were formulated to be adequate in all nutrients relative to the NRC [67] recommendation for each specific pig weight category over the 5 wk period, and included 30% DDGS during each phase of growth. *TIME EFFECT*: In the starter experiment, digestibility of GE, N, carbon (**C**), sulfur (**S**), ADF, NDF and ether extract (**EE**) increased from week-1 to week-5 suggesting that the gastrointestinal tract of the 12 kg pig adapts to dietary fiber from DDGS and nutrient digestibility improves with continuous feeding over time. This finding is consistent with the increased ability of the digestive system in growing pigs to digest nutrients (especially fiber) with increasing age. In contrast, nutrient digestibility did not improve from week-1 to week-5 in finishing pigs. FEED *ADDITIVE EFFECT:* While the results of this research indicate that some of the feed additive products evaluated had variable, but small effects on nutrient digestibility (Tables 3 and 4), none of the products were effective in improving starter and finishing pig growth performance (Table 5) when fed nutritionally adequate corn-soy diets containing 30% DDGS. Many of the enzyme/additive products evaluated in this study contained ingredients that should have been effective in for improving energy/fiber digestibility in 30% DDGS diets. We did not, however, confirm the specified enzyme/active ingredient activity for these additives as it may be possible that they did not contain enough activity to provide significant improvements in digestibility for many of the nutrients evaluated. In addition, because these diets were formulated to meet the nutrient needs of pigs in each growth phase evaluated, the improvements or decreases in nutrient digestibility that did occur were too small to influence overall pig performance.

**Table 3:** **Apparent total tract digestibility (%) of starter pigs fed exogenous feed additives**^**1**^

**Treatment**^**2**^	**GE**	**N**	**C**	**S**	**P**	**ADF**	**NDF**	**EE**
Control	79.2	79.9	79.9	78.5	60.1	40.1	36.6	64.2
Allzyme	76.5	77.6	77.4	77.5	55.6	30.6	27.3	61.5
*P value*^*3*^	*0.01*	*0.01*	*0.01*	*0.17*	*0.01*	*0.01*	*0.01*	*0.14*
BactoCel	80.0	80.4	80.3	80.1	59.8	39.4	39.3	64.9
*P value*^*3*^	*0.14*	*0.55*	*0.42*	*0.03*	*0.79*	*0.76*	*0.15*	*0.66*
BioPlus2B	79.5	80.3	80.0	79.6	58.7	37.7	35.0	65.0
*P value*^*3*^	*0.59*	*0.64*	*0.85*	*0.17*	*0.24*	*0.31*	*0.39*	*0.64*
Econase	78.3	78.7	79.1	77.0	54.0	35.6	32.5	62.8
*P value*^*3*^	*0.07*	*0.07*	*0.10*	*0.04*	*0.01*	*0.06*	*0.03*	*0.45*
Hemicel	78.9	79.0	79.6	79.0	59.5	36.3	33.4	65.5
*P value*^*3*^	*0.53*	*0.17*	*0.48*	*0.49*	*0.60*	*0.12*	*0.09*	*0.45*
Porzyme	79.0	79.4	79.7	78.8	58.4	36.3	33.2	64.9
*P value*^*3*^	*0.67*	*0.47*	*0.61*	*0.66*	*0.16*	*0.13*	*0.07*	*0.67*
Releezyme	76.9	77.4	77.7	77.3	56.1	30.0	29.9	61.1
*P value*^*3*^	*0.01*	*0.01*	*0.01*	*0.09*	*0.01*	*0.01*	*0.01*	*0.08*
Rovabio	80.0	80.7	80.7	79.9	59.5	38.1	36.5	64.4
*P value*^*3*^	*0.12*	*0.25*	*0.14*	*0.06*	*0.61*	*0.39*	*0.97*	*0.88*
Roxazyme	79.6	81.1	80.3	79.9	59.1	38.8	39.1	63.3
*P value*^*3*^	*0.40*	*0.10*	*0.42*	*0.06*	*0.38*	*0.58*	*0.16*	*0.61*
XPC yeast	79.6	80.1	80.3	79.4	57.9	39.0	36.4	65.9
*P value*^*3*^	*0.40*	*0.81*	*0.46*	*0.26*	*0.06*	*0.63*	*0.95*	*0.33*
MODEL								
*P value*^*4*^	*0.01*	*0.01*	*0.01*	*0.01*	*0.01*	*0.01*	*0.01*	*0.08*
SE^4^	0.35	0.48	0.34	0.52	0.80	1.714	1.318	1.221
Wk-1^5^	76.9	76.0	77.6	75.4	55.3	31.4	28.5	70.6
Wk-3	79.2	80.1	79.8	79.3	58.9	36.2	35.8	61.9
Wk-5	80.5	82.4	81.2	81.8	60.0	42.0	39.1	59.4
*P value*^*6*^	*0.01*	*0.01*	*0.01*	*0.01*	*0.01*	*0.01*	*0.01*	*0.01*
SE^6^	0.18	0.25	0.18	0.27	0.42	0.93	0.69	0.64

^1^ Apparent total tract digestibility calculated using indirect marker methodology. There were 16 to 18 individually fed pigs per dietary treatment [66].

^2^ Allzyme SSF, 500 mg/kg (Alltech, Lexington, KY); BactoCel, 110 mg/kg (Lallemand Animal Nutrition, Milwaukee, WI); BioPlus 2B, 500 mg/kg (Chr. Hansen, Milwaukee, WI); Econase XT25, 150 mg/kg (AB Enzymes, Darmstadt, Germany); Hemicel, 500 mg/kg (ChemGen Corp., Gaithersburg, MD); Porzyme 9302, 250 mg/kg (Danisco Animal Nutrition, Marlborough, UK); Reelezyme 4 M, 500 mg/kg (Prince Agri Products Inc., Quincy, IL); Rovabio AP10, 500 mg/kg (Adisseo, Antony, France); Roxazyme G2G, 220 mg/kg (DSM Nutritional Products Inc., Parsippany, NJ); XPC Yeast, 2,000 mg/kg (Diamond V Mills Inc., Cedar Rapids, IA).

^3^^′^P value^′^ represents comparison of the feed additive to the control diet only.

^4^ Model P and SE value for overall diet effect.

^5^ Initial, wk-1, wk-3, and wk-5 BW of 11.88, 13.96, 23.23, and 33.26 kg, respectively.

^6^ Model P and SE value for week.

**Table 4:** **Apparent total tract digestibility (%) of finisher pigs fed exogenous feed additives**^**1**^

**Treatment**^**2**^	**GE**	**N**	**C**	**S**	**P**	**ADF**	**NDF**	**EE**
Control	81.4	83.8	82.3	82.7	39.0	52.9	42.1	46.5
Allzyme	82.1	84.2	83.00	83.3	46.7	56.6	46.9	48.1
*P value*^*3*^	*0.27*	*0.61*	*0.29*	*0.38*	*0.01*	*0.08*	*0.08*	*0.41*
BactoCel	80.8	82.3	82.0	82.4	37.1	50.1	39.5	49.6
*P value*^*3*^	*0.40*	*0.05*	*0.57*	*0.73*	*0.36*	*0.19*	*0.34*	*0.11*
BioPlus2B	81.7	83.2	82.7	82.6	39.1	56.3	45.4	38.6
*P value*^*3*^	*0.58*	*0.46*	*0.49*	*0.91*	*0.96*	*0.10*	*0.23*	*0.01*
Econase	80.8	82.7	81.8	83.1	39.6	50.8	42.0	46.7
*P value*^*3*^	*0.40*	*0.15*	*0.45*	*0.55*	*0.75*	*0.33*	*0.95*	*0.82*
Hemicel	80.7	82.8	81.6	82.4	37.1	48.3	37.4	44.3
*P value*^*3*^	*0.30*	*0.20*	*0.27*	*0.74*	*0.37*	*0.03*	*0.08*	*0.25*
Porzyme	79.4	80.9	80.4	80.1	33.0	43.8	34.0	44.4
*P value*^*3*^	*0.01*	*0.01*	*0.01*	*0.01*	*0.01*	*0.01*	*0.01*	*0.28*
Releezyme	79.5	80.7	80.4	79.9	33.0	50.0	35.4	38.1
*P value*^*3*^	*0.01*	*0.01*	*0.01*	*0.01*	*0.01*	*0.18*	*0.02*	*0.01*
Rovabio	81.3	83.7	82.3	82.8	36.4	52.7	43.5	45.5
*P value*^*3*^	*0.98*	*0.92*	*0.96*	*0.88*	*0.20*	*0.93*	*0.62*	*0.62*
Roxazyme	80.9	81.9	81.7	81.9	37.4	49.8	38.1	49.9
*P value*^*3*^	*0.45*	*0.12*	*0.35*	*0.27*	*0.45*	*0.15*	*0.14*	*0.08*
XPC yeast	80.1	82.5	81.1	82.1	35.6	50.1	38.4	43.1
*P value*^*3*^	*0.05*	*0.10*	*0.05*	*0.36*	*0.09*	*0.19*	*0.18*	*0.08*
MODEL								
*P value*^*4*^	*0.01*	*0.01*	*0.01*	*0.01*	*0.01*	*0.01*	*0.01*	*0.01*
SE^4^	0.45	0.55	0.45	0.47	1.45	1.50	1.95	1.38
Wk-1^5^	80.6	82.3	81.5	81.7	38.6	50.7	40.1	45.3
Wk-3	80.8	82.5	81.8	82.3	37.4	51.7	40.5	44.9
Wk-5	81.0	83.0	82.0	82.3	36.9	50.8	40.2	44.8
*P value*^*6*^	*0.43*	*0.17*	*0.39*	*0.17*	*0.78*	*0.62*	*0.96*	*0.89*
SE^6^	0.24	0.30	0.24	0.25	0.26	0.80	1.04	0.73

^1^ Apparent total tract digestibility calculated using indirect marker methodology. There were 8 individually fed pigs per dietary treatment [66].

^2^ Allzyme SSF, 500 mg/kg (Alltech, Lexington, KY); BactoCel, 110 mg/kg (Lallemand Animal Nutrition, Milwaukee, WI); BioPlus 2B, 500 mg/kg (Chr. Hansen, Milwaukee, WI); Econase XT25, 150 mg/kg (AB Enzymes, Darmstadt, Germany); Hemicel, 500 mg/kg (ChemGen Corp., Gaithersburg, MD); Porzyme 9302, 250 mg/kg (Danisco Animal Nutrition, Marlborough, UK); Reelezyme 4 M, 500 mg/kg (Prince Agri Products Inc., Quincy, IL); Rovabio AP10, 500 mg/kg (Adisseo, Antony, France); Roxazyme G2G, 220 mg/kg (DSM Nutritional Products Inc., Parsippany, NJ); XPC Yeast, 1,000 mg/kg (Diamond V Mills Inc., Cedar Rapids, IA).

^3^^′^P value^′^ represents comparison of the feed additive to the control diet only.

^4^ Model P and SE value for overall diet effect.

^5^ Initial, wk-1, wk-3, and wk-5 BW of 98.40, 104.90, 119.52, and 132.20 kg, respectively.

^6^ Model P and SE value for week.

**Table 5:** **Performance of pigs fed exogenous feed additives**^**1**^

	**Starter, 12 to 33 kg BW**			**Finisher, 98 to 132 kg BW**		
**Treatment**^**2**^	**ADG, kg**	**ADFI, kg**	**G:F**	**ADG, kg**	**ADFI, kg**	**G:F**
Control	0.640	1.126	0.572	0.999	3.032	0.333
Allzyme	0.651	1.140	0.574	0.961	3.118	0.311
BactoCel	0.615	1.083	0.568	1.007	3.084	0.328
BioPlus2B	0.645	1.162	0.559	0.988	3.179	0.315
Econase	0.653	1.133	0.578	1.051	3.240	0.325
Hemicel	0.629	1.149	0.551	0.933	3.239	0.292
Porzyme	0.642	1.131	0.570	0.979	3.077	0.318
Releezyme	0.639	1.109	0.579	0.983	3.115	0.311
Rovabio	0.648	1.148	0.565	0.906	2.985	0.302
Roxazyme	0.638	1.100	0.583	0.975	3.084	0.321
XPC yeast	0.653	1.157	0.568	0.862	2.930	0.294
	MODEL					
*P value*	*0.87*	*0.70*	*0.72*	*0.60*	*0.90*	*0.56*
SE	0.016	0.030	0.011	0.057	0.141	0.014

^1^ Performance over the 5-wk period. There were 16–18 and 8 individually fed pigs per treatment in the starter and finisher phase, respectively [66].

^2^ Allzyme SSF, 500 mg/kg (Alltech, Lexington, KY); BactoCel, 110 mg/kg (Lallemand Animal Nutrition, Milwaukee, WI); BioPlus 2B, 500 mg/kg (Chr. Hansen, Milwaukee, WI); Econase XT25, 150 mg/kg (AB Enzymes, Darmstadt, Germany); Hemicel, 500 mg/kg (ChemGen Corp., Gaithersburg, MD); Porzyme 9302, 250 mg/kg (Danisco Animal Nutrition, Marlborough, UK); Reelezyme 4 M, 500 mg/kg (Prince Agri Products Inc., Quincy, IL); Rovabio AP10, 500 mg/kg (Adisseo, Antony, France); Roxazyme G2G, 220 mg/kg (DSM Nutritional Products Inc., Parsippany, NJ); XPC Yeast, 2,000 mg/kg starter or 1,000 mg/kg finisher (Diamond V Mills Inc., Cedar Rapids, IA).

Unfortunately, results of studies where there are no effects of supplemental enzymes on pig growth performance often are not published in the scientific literature, leading to a publication bias in the information being available to pork producers, swine nutritionists, and other pork industry professionals.

### Phytase alone or in combination with other enzymes

The impact of dietary phytase supplementation on the digestibility of energy has not been consistent. While most studies [68-72] have observed no impact of phytase on energy digestibility, others [73-76] have reported positive effects. Recent results from Kerr et al. [77] were also inconclusive, suggesting that if there is an effect of phytase on energy digestibility, it is relatively small in magnitude and highly variable.

Data relative to the impact of phytase, with or without other enzymes, on nutrient (and energy) digestibility is lacking. Olukosi et al. [78] supplemented diets comprised of corn, wheat midds, soybean meal, and canola meal with either phytase or an enzyme cocktail (xylanase, amylase, and protease) alone, or in combination, and fed them to 10 to 23 kg pigs (Table 6). These data suggest that even though phytase improved pig gain and feed efficiency, addition of the enzyme cocktail, alone or in combination with phytase, had no impact on pig performance. Neither the addition of phytase nor the enzyme cocktail, alone or in combination, had any consistent effect on DM, energy, or N digestibility, but each improved P digestibility. The effects, however, were not additive. In an additional experiment using wheat to replace corn in the diet (23 to 52 kg BW, 42 d trial), there were no effects of phytase or xylanase (500 U and 4,000 U/kg, respectively) on pig performance, or on N and energy digestibility [78]. Phytase, but not xylanase, improved phosphorus digestibility as one would expect from an enzyme that releases phosphate.

**Table 6:** **Growth performance and apparent total tract digestibility of 10 to 23 kg pigs receiving phytase, or a cocktail of xylanase, amylase, and protease**^**1**^

	**Pig performance**			**Apparent total tract digestibility,%**			
**Dietary treatment**	**ADG, g**	**ADFI, g**	**G:F, g:kg**	**DM**	**GE**	**N**	**P**
Negative control (NC)	398	1140	363	80.2	79.8	80.1	38.3
NC + Phytase^2^	483	1070	457	80.1	78.1	80.2	49.9
NC + Enzyme^3^	393	1050	380	82.3	80.1	81.2	48.3
NC + Ph + En	479	1210	415	80.0	79.0	80.0	51.1
SEM	10.4	30	13.7	0.20	0.43	0.43	0.87

^1^ There were 4 replicate pens each of barrows and gilts (1 pig/pen) in the 28 d trial.

^2^ Phytase was added at the rate of 500 phytase units/kg diet [78].

^3^ Cocktail of 400 U of xylanase, 4,000 U of amylase, and 2,500 U of protease per kg of diet.

Results from experiments evaluating the impact of phytase, with or without other enzymes, on nutrient (and energy) digestibility in diets containing DDGS are also lacking and inconsistent. While addition of 500 units phytase improved P digestibility in diets containing 20% DDGS in starter or finisher pigs, it did not improve DM digestibility [79,80]. In contrast, Lindemann et al. [81] reported that 64 to 123 kg pigs fed diets containing 20% DDGS supplemented with 250 or 500 U/kg phytase exhibited greater DM, energy, and N digestibility than unsupplemented pigs, but there were no further improvements in fecal DM, energy or N digestibility when xylanase was added in addition to the phytase addition.

### Energy and Fiber in Corn Co-products

Gross energy in DDGS averages 5,434 kcal/kg DM and is greater than the concentration of GE in corn (Table 7) [82]. However, the digestibility of energy, measured as a percentage of GE, is lower in DDGS than in corn [82]. The digestible energy (**DE**) and metabolizable energy (**ME**) content of DDGS is 4,140 and 3,897 kcal/kg DM, respectively [83], which are similar to the DE and ME content in corn (Table 7). The net energy value of DDGS has not been determined, but research is currently underway to measure these values.

**Table 7:** **Concentration of energy in corn and 10 sources of corn distillers dried grains with solubles (DDGS) fed to growing pigs**^**1**^

		**DDGS**			
**Item**	**Corn**	**Average**	**SD**	**Lowest value**	**Highest value**
GE, kcal/kg DM	4,496	5,434	108	5,272	5,592
ATTD^2^ of energy,%	90.4	76.8	2.73	73.9	82.8
DE, kcal/kg DM	4,088	4,140	205	3,947	4,593
ME, kcal/kg DM	3,989	3,897	210	3,674	4,336

^1^ Data from Pedersen et al. [83] as adapted from Stein and Shurson [82].

^2^ ATTD = apparent total tract digestibility.

Since most of the starch in corn has been converted to ethanol, DDGS contains approximately 35% insoluble and 6% soluble dietary fiber [82] (Table 8). Likewise, most corn co-products have a high amount of insoluble fiber which can be observed by comparing the relatively similar TDF and NDF concentrations in these co-products [84] (Table 9). Furthermore, corn ″fiber″ has a large hemicellulose component as defined by the difference between NDF and ADF. These results are similar to those reported by Leathers [85], where the corn fiber composition from six studies representing different geographic regions showed that hemicellulose is the predominant constituent in corn fiber, followed by xylose (Table 10).

**Table 8:** **Concentration of carbohydrates and apparent total tract digestibility (ATTD) of dietary fiber in corn distillers dried grains with solubles**^**1**^

**Item**	**Average**	**Low value**	**High value**	**SD**
Starch, total,%	7.3	3.8	11.4	1.4
Starch, soluble,%	2.6	0.5	5.0	1.2
Starch, insoluble,%	4.7	2.0	7.6	1.5
ADF,%	9.9	7.2	17.3	1.2
NDF,%	25.3	20.1	32.9	4.8
Insoluble TDF,%	35.3	26.4	38.8	4.0
Soluble TDF,%	6.0	2.36	8.54	2.1
TDF,%	42.1	31.2	46.3	4.9
ATTD of TDF,%	43.7	23.4	55.0	10.2

^1^ N = 46 for data on starch, ADF, and NDF; n = 8 for data on insoluble, soluble, and total dietary fiber [82].

**Table 9:** **Analyzed composition of corn co-products, DM basis**^**1**^

**Item**	**DDGS (WI)**	**DDGS (IA)**	**DDGS (SD)**	**RO-DDGS (SD)**	**DDGS (BPX)**	**Drum-DDGS (MN)**	**Microwave-DDGS (MN)**	**Dried solubles**	**Gluten feed**
Crude protein	29.62	29.65	31.94	34.74	29.49	32.69	34.12	23.75	24.29
Starch	7.85	3.47	6.24	3.04	4.94	2.12	1.05	6.34	12.57
Crude fiber	7.05	7.76	7.56	8.69	7.95	7.93	8.35	0.08	8.56
TDF	30.34	38.14	35.69	37.20	35.90	35.38	43.18	16.07	40.07
NDF	34.61	40.13	40.12	50.96	33.41	44.87	49.12	2.33	42.66
ADF	11.25	10.55	14.42	15.82	8.62	13.16	14.66	0.49	9.90
Cellulose	10.64	10.12	11.72	12.72	8.21	11.95	13.37	0.79	9.17
Lignin	1.21	1.06	3.16	3.49	1.00	1.72	1.92	0.31	1.05
**Item**	**DHDG corn**	**Dehy corn germ**	**Corn germ meal**	**Bran**	**Bran + solubles**	**Gluten meal**	**HP-DDG (MOR)**	**HP-DDG (Poet)**	**HP-DDG (ICM)**
Starch	87.96	25.00	15.29	23.25	25.73	11.08	0.51	7.30	5.10
Crude fiber	0.60	4.87	10.69	11.54	4.80	1.44	8.14	9.42	7.87
TDF	2.61	24.78	47.76	53.60	26.65	9.24	28.80	31.28	36.75
NDF	4.27	27.37	61.05	56.86	25.21	12.25	43.52	32.00	51.09
ADF	0.49	6.13	12.49	13.14	5.35	7.57	25.42	12.61	15.11
Cellulose	0.77	5.21	11.71	12.78	5.38	5.95	22.55	12.05	14.25
Lignin	0.33	1.28	1.22	0.89	0.55	2.24	3.40	0.95	1.44

^1^Abbreviations: TDF, total dietary fiber; NDF, neutral detergent fiber; ADF, acid detergent fiber; RO-DDGS, reduced oil-DDGS; drum- or microwave-dried DDGS; DHDG, dehulled-degermed; HP-DDG, high protein dried distillers grains. Abbreviations within brackets ( ) refers to the state or company where the product was obtained [84].

**Table 10:** Major components of corn fiber

	**Geographic location**					
**Component**	**A**	**B**	**C**	**D**	**E**	**F**
Starch	22	11	18	22	20	23
Hemicellulose	40	53	32	47	29	39
Xylose	24	25	20	28	18	19
Arabinose	16	18	10	19	11	11
Cellulose	12	18	24	ND	14	ND
Protein	12	11	ND	ND	11	12

ND = not determined [85].

The apparent total tract digestibility of dietary fiber in DDGS averages 43.7%, but ranges from 23% to 55%. This variation in fiber digestibility is believed to influence digestibility of energy in DDGS. Apparent ileal digestibility and total tract digestibility of dietary fiber in DDGS is higher than in corn, and are presumed to be improved as a result of the processing and fermentation processes used in ethanol plants [86]. However, less than 50% of total dietary fiber is fermented over the entire digestive tract, indicating that more than 50% passes through pigs without being fermented [86]. As a result, there is a significant amount of non-fermented carbohydrate in DDGS that could potentially be utilized to a greater extent if appropriate exogenous enzymes can be developed to enhance the utilization of these substrates in DDGS diets.

Consequently, when evaluating the effectiveness of exogenous enzymes, the composition of ″fiber″ must be considered in order for energy and nutrient digestibility to potentially be improved. This is clearly demonstrated by Li et al., [61] who evaluated the effectiveness of adding β-glucanase to a broad range of diets, differing largely in β-glucan content. Their data showed that supplementation of β-glucanase had no effect on energy digestibility in wheat-, corn-, or rye-soybean meal diets, but did improve energy digestibility in barley-soybean meal diets (Table 11), which reflected the dietary differences in β-glucan concentrations.

**Table 11:** Effect of β-glucanase supplementation on energy digestibility

	**Diet composition,%**			**β-glucanase supplementation,%**			
**Diet**	**NDF**	**ADF**	**β-glucans**	**0**	**0.05**	**0.10**	**0.20**
Barley-SBM	8.4	2.3	3.2	85.2	87.8	86.4	88.5
Wheat-SBM	7.9	2.5	0.8	86.8	88.1	88.4	88.4
Corn-SBM	8.1	1.9	0.3	85.8	84.4	83.8	85.7
Rye-SBM	7.4	2.1	0.7	87.2	88.0	88.1	87.1

[61].

### Enzyme activity and substrates

It is clear that there needs to be an improved characterization of fibrous components in all feedstuffs [2]. Likewise, there needs to be some agreement on key enzyme activities and analysis of these activities so that a scientific evaluation of enzyme/feed additive products can be achieved. Lastly, a better understanding of enzyme-substrate relationships combined with an improved understanding of gastrointestinal physiology in relation to enzyme-substrate will improve our understanding of when exogenous feed enzymes will likely have a significant, positive response, with a listing of key enzymes listed in Table 12.

**Table 12:** Key enzyme activity and associative substrate

**Activity**	**Substrate**
Xylanase	Arabino-xylose (NSP)
β-Glucanase	β-Glucans (NSP)
Mannase	Oligo-mannans (Mannose)
α-Galactosidase	α-Galactosyl moieties
Cellulase	Cellulose (α-glucans)
Pectanase	Pectins (NSP)
Amylase	Amylose
Protease	Proteins
Phytase	Phytic acid

## Conclusions

Application of enzymes in an effort to improve nutrient digestibility of plant-based feed ingredients for swine and poultry has been studied for decades. However, with a large diversity and concentration of chemical characteristics existing among plant-based feed ingredients, as well as interactions among constituents within feed ingredients and diets, improvements in nutrient digestibility and pig performance from adding exogenous enzymes to growing pig diets depends on understanding these characteristics in relation to enzyme activity. Essentially, the enzyme must match the target substrate(s), there may need to be a ^′^cocktail^′^ of enzymes to effectively breakdown the complex matrixes of fibrous carbohydrate structures, and there must be some negative role that these substrates have on nutrient digestibility or voluntary feed intake. With the inverse relationship between fiber content and energy digestibility being well described for several feed ingredients, it is only logical that development of enzymes that degrade fiber, and thereby improve energy digestibility or voluntary feed intake will have a greater likelihood to be beneficial to improve fiber utilization in swine, both metabolically and economically. The results of an unpublished study by the authors suggests that although some of the enzyme/additive products evaluated had variable, but small effects on nutrient digestibility, none of these products were effective in improving starter and finishing pig growth performance when fed nutritionally adequate corn-soy diets containing 30% DDGS.

## Abbreviations

ADF: Acid detergent fiber; ADG: Average daily gain; ADFI: Average daily feed intake; AID: Apparent ileal digestibility; ATTD: Apparent total tract digestibility; C: Carbon; CP: Crude protein; DE: Digestible energy; DDGS: Dried distillers grains with soluble; DM: Dry matter; EE: Ether extract; GE: Gross energy; GF: Gain to feed ratio; ME: Metabolizable energy; N: Nitrogen; NDF: Neutral detergent fiber; NSP: Nonstarch polysaccharides; P: Phosphorus; S: Sulfur; TDF: Total dietary fiber; TTD: Total-tract digestibility; VFA: Volatile fatty acids.

## Competing interests

The authors declared that they have no competing interests.

## Authors’ contributions

BJK and GCS co-wrote this review and any internal research reported was jointly designed and interpreted. Both authors have read and approved the manuscript.

## Authors’ information

Brian J. Kerr, Ph.D., is a Lead Scientist/Animal Scientist for the USDA Agricultural Research Service, with expertise in digestibility, nutrient utilization, and evaluation of alternative feed ingredients. Gerald C. Shurson, Ph.D., is a Professor of Animal Science at the University of Minnesota with expertise in nutrition and the use of alternative feed ingredients in swine production.

Mention of a trade name, proprietary product, or specific equipment does not constitute a guarantee or warranty by the USDA or the University of Minnesota and does not imply approval to the exclusion of other products that may be suitable. The USDA is an equal opportunity provider and employer.
